# Small Renal Masses: Developing a Robust Radiomic Signature

**DOI:** 10.3390/cancers15184565

**Published:** 2023-09-14

**Authors:** Michele Maddalo, Lorenzo Bertolotti, Aldo Mazzilli, Andrea Giovanni Maria Flore, Rocco Perotta, Francesco Pagnini, Francesco Ziglioli, Umberto Maestroni, Chiara Martini, Damiano Caruso, Caterina Ghetti, Massimo De Filippo

**Affiliations:** 1 Medical Physics Unit, University Hospital of Parma, 43126 Parma, Italy; mmaddalo@ao.pr.it (M.M.); amazzilli@ao.pr.it (A.M.); cghetti@ao.pr.it (C.G.); 2 Department of Medicine and Surgery, Section of Radiology, University of Parma, Via Gramsci 14, 43126 Parma, Italy; lorenzo.bertolotti@unipr.it (L.B.); rocco.perotta@unipr.it (R.P.); martinic@ao.pr.it (C.M.); 3 Porretta Terme Hospital, AUSL Bologna, 40046 Porretta Terme, Italy; andreagiovanniflore@gmail.com; 4 Diagnostic Department, Parma University Hospital, Via Gramsci 14, 43126 Parma, Italy; fpagnini@ao.pr.it; 5 Department of Urology, Parma University Hospital, Via Gramsci 14, 43126 Parma, Italy; fziglioli@ao.pr.it (F.Z.); umaestroni@ao.pr.it (U.M.); 6 Radiology Unit, Department of Medical Surgical Sciences and Translational Medicine, Sant’Andrea University Hospital, Sapienza-University of Rome, 00100 Rome, Italy

**Keywords:** small renal masses, radiomics, malignant, benign, characterization, kidney cancer, oncocytoma, renal cell carcinoma

## Abstract

**Simple Summary:**

Renal cell carcinoma (RCC) is frequently diagnosed at the early localized stage as an incidental finding (about 60% of cases). Imaging procedures (ultrasound, CT, MRI) represent the only way to diagnose RCC, but they are not always reliable for the discrimination between malignant and benign tumors, in particular when the renal mass is small (<4 cm) because they demonstrate low diagnostic specificity. The quantitative analysis of contrast-enhanced CT in venous phase using radiomics could provide additional information for the accurate characterization of small renal masses (SRMs).

**Abstract:**

(1) Background and (2) Methods: In this retrospective, observational, monocentric study, we selected a cohort of eighty-five patients (age range 38–87 years old, 51 men), enrolled between January 2014 and December 2020, with a newly diagnosed renal mass smaller than 4 cm (SRM) that later underwent nephrectomy surgery (partial or total) or tumorectomy with an associated histopatological study of the lesion. The radiomic features (RFs) of eighty-five SRMs were extracted from abdominal CTs bought in the portal venous phase using three different CT scanners. Lesions were manually segmented by an abdominal radiologist. Image analysis was performed with the Pyradiomic library of 3D-Slicer. A total of 108 RFs were included for each volume. A machine learning model based on radiomic features was developed to distinguish between benign and malignant small renal masses. The pipeline included redundant RFs elimination, RFs standardization, dataset balancing, exclusion of non-reproducible RFs, feature selection (FS), model training, model tuning and validation of unseen data. (3) Results: The study population was composed of fifty-one RCCs and thirty-four benign lesions (twenty-five oncocytomas, seven lipid-poor angiomyolipomas and two renal leiomyomas). The final radiomic signature included 10 RFs. The average performance of the model on unseen data was 0.79 ± 0.12 for ROC-AUC, 0.73 ± 0.12 for accuracy, 0.78 ± 0.19 for sensitivity and 0.63 ± 0.15 for specificity. (4) Conclusions: Using a robust pipeline, we found that the developed RFs signature is capable of distinguishing RCCs from benign renal tumors.

## 1. Introduction

Renal cell carcinoma is a malignant tumor with a prevalence rate of 3% in Europe, ranking eighth among the most frequent cancers in the general population in Italy [1,2]. In the last decades, the number of diagnoses of renal carcinoma has progressively risen due to both the improvement of imaging techniques and their increasing use in clinical practice [3]. The diagnosis of these tumors is increasingly frequent as an incidental finding (about 60% of cases), with an increasing number of lesions diagnosed at an early localized stage [2,4]. The diagnosis of renal cell carcinoma (RCC) can only be performed by imaging (ultrasound, CT, MRI), however, imaging methods are also not always reliable in distinguishing between benign neoplasms (oncocytoma and angiomyolipoma are among the most frequent) and malignant cancer (in particular, clear cell carcinoma, papillary carcinoma and chromophobe carcinoma) [5]. Diagnostic uncertainty in the differentiation between benign and malignant cases is particularly high when dealing with small renal masses (SRMs), i.e., a renal mass that has a diameter of less than 4 cm, as the specificities of contrast-enhanced CT and MRI for predicting RCC are as low as 44.4 and 33.3%, respectively [6,7].

Histological characterization of the mass by renal mass biopsy (RMB) could be a useful tool for correct diagnosis to avoid the potential morbidity associated with the overtreatment of SRMs. Nonetheless, RMB is not risk-free, as bleeding and tumor seeding [8] could occur; concerning complications of RMB, the most common are hematoma (4.9%) and clinically significant pain (1.2%), but gross hematuria (1.0%), pneumothorax (0.6%) and hemorrhage (0.4%) have also been reported in some patients [9].

It is important to also note that pre-procedural biopsies are non-diagnostic in a percentage as high as 15–22% of cases [10,11], with a median 29% nondiagnostic rate in patients presenting with cystic lesions [8]. Another important predictor is tumor size; the smaller the lesion, the more likely it is to have a nondiagnostic biopsy; SRMs have high false negative rates, with a low reported negative predictive value of 60%. Furthermore, benign biopsy histology cannot rule out malignancy in the rest of the tumor, particularly in chromophobe varieties. A definitive benign diagnosis may be inferred from an RMB when the pathology is consistent with angiomyolipoma, metanephric adenoma or focal infection. A biopsy specimen showing non-diagnostic or non-malignant findings must be considered with caution, and surveillance imaging, repeat biopsy or surgery are currently recommended [10].

The accurate characterization of SRMs, therefore, becomes fundamental for correct diagnostic classification, defining the best therapeutic procedure for the patient, avoiding unnecessary surgery in the case of benign renal masses and expanding the use of percutaneous image-guided minimally invasive ablative treatments of small masses [11,12,13], especially for unfit and comorbid patients with masses < 3 cm, according to European urological guidelines [2].

Novel methods for the characterization of renal masses that make use of radiomics to evaluate tumor characteristics and enhance diagnostic capabilities by extracting quantitative features from medical images are currently under investigation [14,15,16,17,18,19,20]. Among the studies that have questioned the utility of radiomics for the characterization of renal masses, only a few of them have focused on SRMs [10,21,22,23], trying to address the problem of the extremely low specificity that characterizes their radiological evaluation. However, the majority of these studies developed their predictive models on multi-phase contrast-enhanced CT specifically designed for renal mass characterization, not exploring the possibility of a wider applicability of radiomics using venous phase CT where renal masses are incidentally found.

The aim of the present study was to develop a predictive model based on radiomics in order to improve the diagnostic capability of imaging in distinguishing between benign and malignant small renal masses only using venous phase CT acquisitions, which were routinely acquired during exams made for other reasons.

In this way, we developed a radiomic signature that is more extensively applicable and easier to reproduce.

## 2. Materials and Methods

In this retrospective, observational, monocentric study, we selected a cohort of patients, enrolled between January 2014 and December 2020, with a newly diagnosed renal mass smaller than 4 cm (SRM) that later underwent nephrectomy surgery (partial or total) or a tumorectomy with an associated histopathological study of the lesion. The Institutional Review Board approved this study and informed consent was retrieved for enrolled patients when feasible given the retrospective nature of the study and the analysis used anonymous clinical data.

### 2.1. Patients

Adult patients presenting for surgical resection of renal masses at the AOU of Parma were considered for inclusion if contrast-enhanced abdominal CT studies in the venous phase were available. Only renal masses with the largest diameter of 40 mm in any direction were included in this study. Exclusion criteria were diffuse infiltrative renal disease (i.e., lymphoma), CT artifacts that could compromise renal lesion segmentation or acute intralesional complications (e.g., hemorrhage). Patients with lipid-rich angiomyolipomas, identified by the presence of macroscopic fat on CT, were excluded from radiomic evaluation. The effect of the inclusion/exclusion criteria is provided as a flow chart in Supplementary Materials (Figure S1).

Demographics and clinical data were collected and included in a dedicated anonymous database, including the surgical treatment performed. By applying these enrollment criteria, we found 85 patients (range 38–87 years old; 51 men), of whom 51 had malignant histology and 34 had benign histology.

### 2.2. CT Imaging

All patients underwent contrast-enhanced CT with an iodine contrast injection of high concentration (300 mg I/mL, Iomeron 300, Bracco, Italy), a 90–130 mL volume (based on patient weight), and a 3–4 mL/s flow rate. The contrast-enhanced scan was triggered by 150 HU density in the abdominal aorta (at the level of the celiac axis) and the portal venous phase was acquired with a 60 s delay (standard protocol). Both the single portal venous phase and the venous phase of a multi-phase CT were included. The CT scans were acquired using three different CT scanners (Siemens SOMATOM Emotion 6, Siemens SOMATOM Sensation Cardiac 64 and Siemens SOMATOM Definition Flash—Siemens Healthcare, Berlin, Germany) with different acquisition parameters: tube voltage between 100 and 130 kVp, variable values between 0.61 and 0.98 mm for pixel spacing and between 1.5 mm and 2.5 mm for slice thickness; five different values for the reconstruction kernel (B31s, B40s, B20f, B30f, Br32f). The DICOM header of images was analyzed to retrieve the acquisition and reconstruction parameters for a subsequent reproducibility analysis of radiomic features.

### 2.3. Region of Interest (ROI) Detection and Calculation of Radiomic Features 

**Figure 1:**
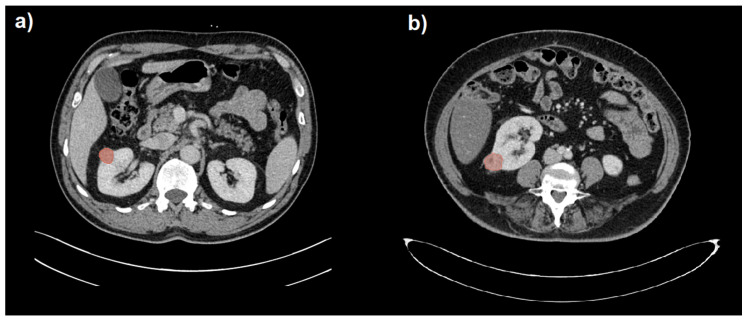
Segmentation (in red) of a benign (**a**) and a malignant (**b**) small renal mass (SRM) hardly distinguishable on portal venous phase CT images.

The CT images and related radiological data were extrapolated from the PACS data archiving system of Parma University Hospital. Images were anonymized before their export. Two readers (R1, a radiologist with 15 years of experience in abdominal imaging; R2, a radiologist with 3 years of experience in abdominal imaging) reviewed the CT scans (blinded to clinical and pathological information). The abdominal CTs of the patients included in the study were then imported into 3D Slicer software version 4.10.2 [24]. The radiologist with 3 years of experience (R2) manually delineated the region of interest (ROI) along the edge of the lesion, layer by layer, on the portal venous phase by manually drawing the tumor boundaries. The ROI was used to delineate the boundary of all planes of the renal mass, including necrosis, cystic degeneration and hemorrhage; however, it did not include normal renal tissue or perirenal fat. The volume of interest (VOI) of the lesion was then automatically generated by the software. Finally, another senior radiologist (R1) examined the outlined results on multiplanar reconstruction (MPR) images. Examples of benign and malignant small renal masses with respective segmentations are depicted in Figure 1. No image preprocessing such as wavelet and LoG transformations was performed on CT images before radiomic analysis, so only RFs belonging to the original type were considered. Subsequently, 108 RFs were extracted from the VOI using the SlicerRadiomics^®^ tool [25]. The extracted RFs included both first-order and subsequent-order features, including shape, first-order, Gray-Level-Co-occurrence-Matrix (GLCM), Gray-Level-Run-Length-Matrix (GLRLM), Gray-Level-Size—Zone-Matrix (GLSZM), Neighboring-Gray-Tone-Difference-Matrix (NGTDM) and Gray-Level-Dependence-Matrix (GLDM) functions.

### 2.4. Radiomic Analysis 

**Figure 2:**
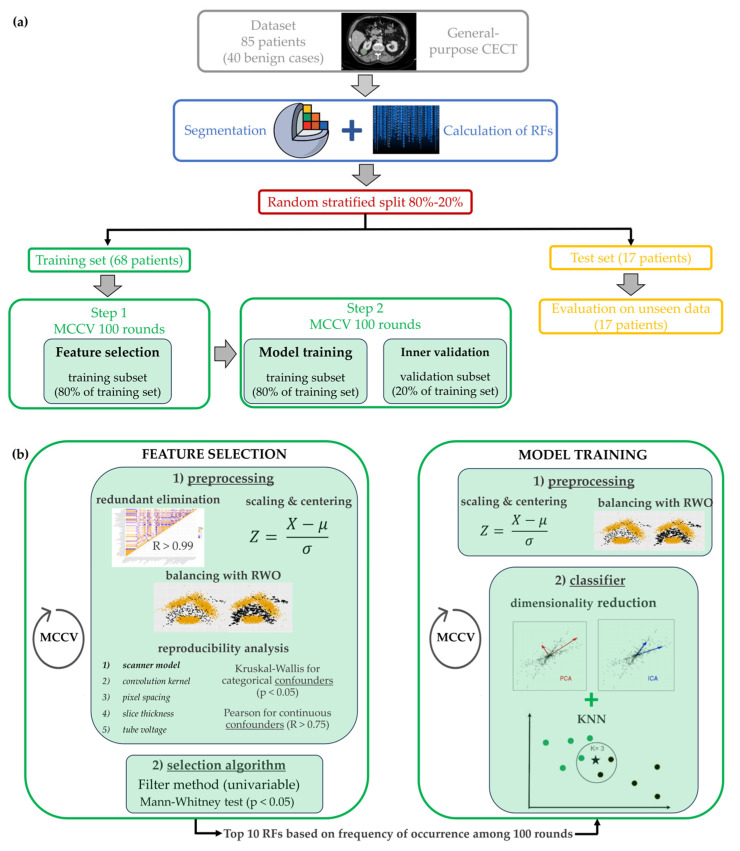
Whole analysis pipeline to develop the radiomics signature, including CT images segmentation, ROI analysis with Slicer Software, features selection and the model training and validation. (**a**) Generic overview of the whole pipeline, from CT image segmentation to model training and testing, (**b**) Detailed focus on the two steps of the machine learning method, i.e., the feature selection and the model training. The employed classifier was kNN: each patient was represented by a point in the feature space and it was classified by the algorithm based on its fist k neighbors and on the Euclidean distance with respect to each of them. In the example of figure (**b**), the patient marked with ★ is compared to the three (k = 3) closer patients which could have either a benign (marked with •) or a malign (marked with •) lesion.

The analysis pipeline is schematized in Figure 2.

Training and test sets were obtained by randomly and blindly splitting the dataset into two parts considering a proportion of 80–20% and balancing with respect to the outcome variable. On the training set, two consecutive Monte Carlo cross-validations (MCCVs) with 100 rounds were implemented using the same proportion of 80–20% and endpoint balancing, one was used for feature selection and one for model training. MCCV systematically repeats (100 times) a random split of the database into training/validation subsets (slightly changing the patients included in both subsets each time), and thus, it allows testing of the stability of the model by evaluating the change in the model performances due to a different inclusion of cases in the training subset. Feature selection and model training represented two independent steps of the analysis (Figure 2). The feature selection step was performed on the training subset (80% of the training set). It ended with a unique subset of selected RFs, which was then passed as input for the model training (second step). In the second step, the model was iteratively trained using different training subsets (80% of the training set) and then applied without modification on the validation subset (20% of the training set) for unbiased evaluation of the model’s performances. During each round of the second step, the models were also evaluated on the test set. The steps of the analysis pipeline are described below in detail.

Feature selection and model training are two independent steps, but they use exactly the same operations on RF values (i.e., the z-score) and on the number of instances (i.e., the oversampling algorithm). In the first step, the preprocessing consists of redundant RF elimination, RF standardization, minority class oversampling and reproducibility analysis. Regarding redundant RF elimination, a cross-correlation matrix between RFs was calculated to detect highly correlated features. RF pairs with a correlation coefficient R > 0.99 were identified as redundant and excluded for later analysis. Then, RFs were scaled and centered using a z-score. Endpoint imbalance was addressed by over-sampling the minority class in the training set with synthetic examples through Random walk oversampling (RWO). RWO attempted to preserve the variance and mean of the minority class. The last step of preprocessing concerns the reproducibility of RFs related to the acquisition and reconstruction parameters. Reproducibility was investigated using the Kruskal Wallis (KW) test for categorical parameters (i.e., scanner model, convolution kernel) and by Spearman correlation for continuous ones (i.e., pixel spacing, slice thickness, tube voltage). An RF was removed from later analysis if it was significantly associated with at least one categorical parameter (*p*-value of KW < 0.05) or was strongly correlated with at least one of the continuous parameters (correlation coefficient R > 0.75). Reproducibility analysis was carried out as a chain of consecutive tests applied in the following order: test n.1—scanner model, test n.2—convolution kernel, test n.3—pixel spacing, test n.4—slice thickness and test n.5—tube voltage. All preprocessing operations were performed on the training subset and then transferred without modification to both the validation subset and test set, except for RWO, which was not applied at all. 

Most predictive RFs were found using the nonparametric Mann-Whitney (MW) test, which acted as a univariable feature selection filter. RFs were selected if the *p*-value of the test was less than a significance level of 0.05. A Monte Carlo cross-validation of 100 iterations was implemented for the FS step. During each iteration of MCCV, the RFs were scored if the MW *p*-value was less than 0.05. Finally, the 10 RFs that had higher cumulative scores (i.e., the sum of scores of all MCCV rounds) were selected: they were fixed as input for the second MCCV of model training.

In the second step, after the preprocessing operations of RFs standardization and minority class oversampling, the training algorithm consisted of a k-nearest neighbors (kNN) classifier preceded by a dimensionality reduction technique; that is, principal component analysis (PCA) or independent component analysis (ICA). The tuning parameters were the technique used for dimensionality reduction, the number of components (d) and the number of neighbors (k). During the MCCV, the model parameters were kept fixed. The second step was then repeated several times by changing the values of the parameters for optimization. The final model with tuned parameters was identified as the one that maximized the average F1 score across the 100 rounds of MCCV on the validation subset. Test performances were only extracted for the final model. The performance metrics of training (validation subset) and test sets included ROC-AUC, accuracy, sensitivity and specificity, and were expressed as mean values of 100 iterations with associated standard deviations. The analysis pipeline was developed using the R software environment (version 4.0.4). The R packages used were Caret and Imbalance (for the RWO algorithm).

The above methodology was carried out by adhering as much as possible with respect to the Checklist for Artificial Intelligence in Medical Imaging (CLAIM).

## 3. Results

### 3.1. Patients

The study population was composed of fifty-one RCC (thirty-seven clear cell, seven chromophobe and seven papillary), seven lipid-poor angiomyolipoma, twenty-five oncocytoma, and two renal leiomyomas (Table 1).

### 3.2. Radiomic Analysis

**Figure 3:**
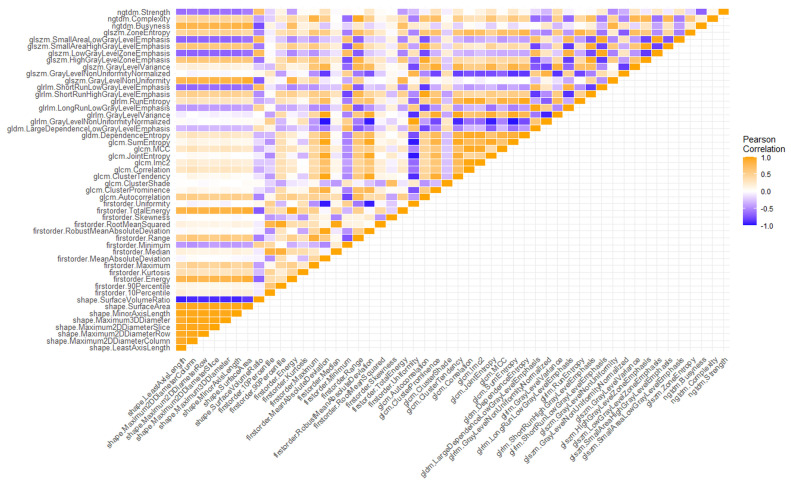
Heatmap showing the correlation matrix among radiomic features.

Regarding the first step of feature selection, the first available RFs were filtered by correlation analysis, reproducibility analysis and the feature selection algorithm itself. Regarding the correlation matrix, the heatmap of cross-correlation among RFs is depicted in Figure 3. A relevant percentage of redundant RFs, i.e., (26.6 ± 2.8)%, was detected. 

Of the resulting non-redundant RFs, approximately 30% were identified as non-reproducible against the scanner model during test n.1 of the reproducibility analysis. In test n.2, about 4% was removed as they were significantly affected by the convolution kernel. No RFs were found to be unreproducible concerning pixel spacing, slice thickness and tube voltage.

**Figure 4:**
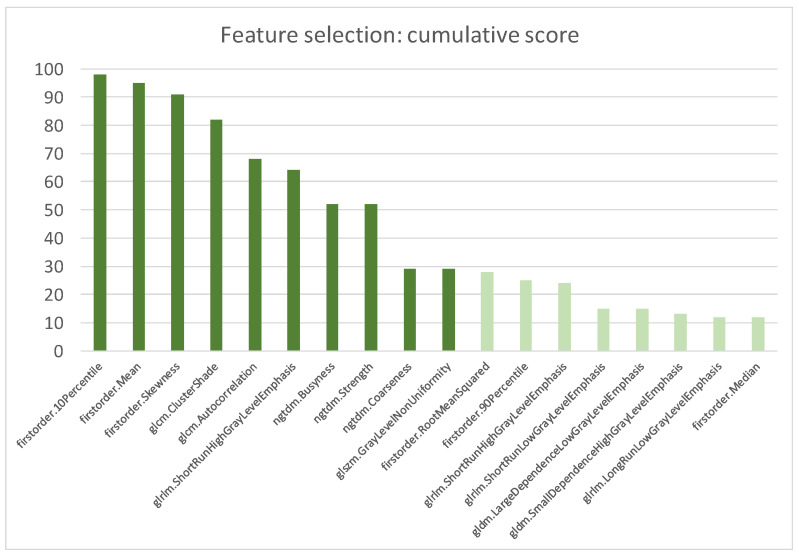
Most scored features after 100 rounds of MCCV. The cumulative score was calculated as the sum of the scores of all MCCV rounds.

After these redundancy and reproducibility analyses, a significantly decreased number of RFs from 107 to (52 ± 8) was passed to the features selection algorithm. At the end of the first step, the scored RFs after 100 rounds of MCCV are reported in Figure 4. 

The top scored RFs included three first-order features (i.e., Ten Percentile, Mean and Skewness) that accounted of the distribution of voxel intensities and seven higher-order features that evaluated the image texture inside the segmented volume. The selected textural parameters described the spatial relationship of voxel intensities (i.e., ClusterShade, Autocorrelation of Gray Level Cooccurrence Matrix), identified homogeneous regions having voxels with same intensities (i.e., ShortRunHighGrayLevelEmphasis of Gray Level Run Length Matrix and GrayLevelNonUniformityNormalized of Gray Level Size Zone Matrix) and quantified difference of intensity between a voxel and its neighborhood (i.e., Busyness, Coarseness and Strength of Neighbouring Gray Tone Difference Matrix).

**Figure 5:**
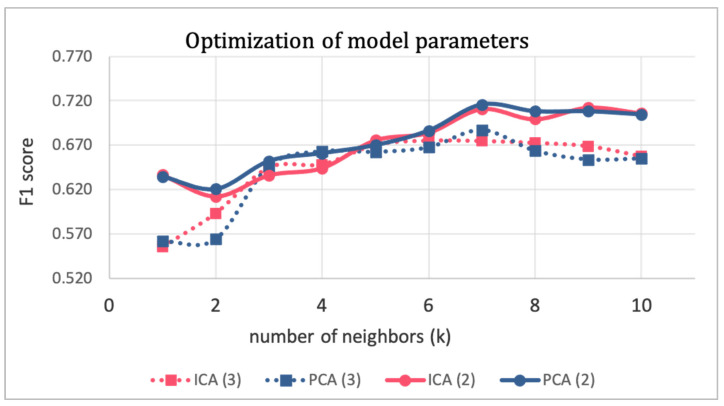
Optimization of model parameters.

The second step was then repeated several times by changing the parameter values to perfect them. The optimization of model parameters is shown in Figure 5, where the F1 score of the validation subset is plotted against the number of neighbors k for different combinations of other parameters (i.e., the technique for dimensionality reduction and the number of dimensions d). To preserve the simplicity and explainability of the model, and to avoid the curse of dimensionality pitfall, d was constrained to values of two and three. Finally, the tuned parameters that maximized the F1 score were PCA with two components and k equal to seven.

Finally, for each round of MCCV, the final model with tuned parameters was evaluated on unseen data of the test set. Performance metrics (mean ± standard deviation) of the final model in the training set (validation subset) and in the test set are reported in Table 2.

The performances in the training and test sets were substantially in agreement. A greater difference was seen for specificity. However, the mean test specificity and the mean training specificity differed by less than one standard deviation of the sample. 

**Figure 6:**
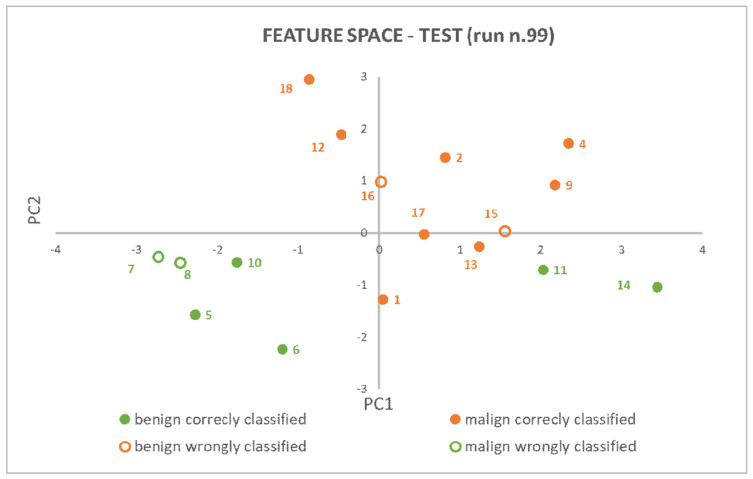
PCA (2) + 7−NN model explainability: patients have been represented as points in a 2D features space and class membership has been proven based on neighbors’ points and their reciprocal distances.

An example of model explainability is depicted in Figure 6, which was extracted from the test results of the rounds that had random seeds equal to 99. It stands for the components space of the features in two dimensions (d = 2), in which the 7−NN algorithm evaluates distances between patients’ pairs (i.e., points in the feature components space) and individuates the seven nearest neighbors.

The tumor histotypes of the 17 patients belonging to the test set and the ones that are mislabelled are reported in Supplementary Materials (Table S1). The data in Table S1 represents a rough/tentative failure analysis of incorrectly classified cases, limited to the influence of the histotype of the success or failure of SRM classification.

The evaluation of methodology adherence of the present study with respect to the Checklist for Artificial Intelligence in Medical Imaging (CLAIM) is provided in Supplementary Materials (Table S2).

## 4. Discussion

The differentiation between benign and malignant renal masses using radiomics represents an innovative field in radiology and oncology; studies in the literature have reported radiomic-based machine learning or deep learning models that successfully predicted the nature of the lesion [15,18,19,21]. In our study, the selected RFs and the identified ML algorithm only obtained from segmentation in the portal venous phase demonstrated good diagnostic accuracy in predicting the malignancy of a renal lesion, both in training sets with an ROC-AUC of 0.79 ± 0.04, an accuracy of 0.75 ± 0.04, a sensitivity of 0.77 ± 0.07 and a specificity of 0.73 ± 0.05 in the final model, and test sets with an ROC-AUC of 0.79 ± 0.12, an accuracy of 0.73 ± 0.12, a sensitivity of 0.78 ± 0.19 and a specificity of 0.63 ± 0.15.

In the clinical context of SRM characterization, the double aim of developing a robust radiomic signature is: (1) to determine which patients have benign SRMs and should not have surgery, as the overtreatment of SRMs yields an unknown survival benefit, can expose patients to psychosocial stressors, perioperative complications and reduced renal function; (2) to allow active surveillance or minimally invasive treatment in patients with small localized malignancies. Even if the accuracy of contrast-enhanced CT and MR in differentiating malignant from benign renal masses is high [26,27], it dramatically decreases when only SRMs are included. By only focusing on small renal masses, the specificity reached by our radiomic signature is higher than the reported specificity of contrast-enhanced MR and CT [6,28] with a comparable sensitivity. In particular, our radiomic signature can better identify both benign and malignant lesions succeeding in the aim of decreasing the overtreatment and of better delineating a malignancy risk stratification and subsequent approach for malignant SRMs. Moreover, these data can be implemented with clinical, deep learning, radiometabolomics, SPECT and transcriptomics data [29,30,31,32,33] to improve performances. Klontzas et al. [32] showed that the radiomics-only performance for distinguishing benign from malignant renal masses was 70%, while the integration of radiomics and metabolomics increased the performance in differentiating malignant lesions (solid, cystic or mixed) to at least 86%. Furthermore, Klontzas et al. [30], by combining the ^99m^Tc Sestamibi uptake with radiomics in distinguishing benign oncocytic neoplasia, increased the diagnostic accuracy and improved positive and negative predictive value. Finally, transcriptomics and radiomics have been combined to assess the prognosis of RCC patients, as mentioned by Tang et al. [29] (C-index: 0.927 and 0.879 for OS- and DFS-predicting, respectively).

The patient cohort in our study showed characteristics consistent with the prevalence of renal carcinomas in the general population. Specifically, there was a male predominance, with males forming 60% of our study population, and the mean age of our study participants was 61 years old, in line with the peak incidence of SRMs occurring between 60 and 70 years. Notably, the proportion of benign renal masses compared to renal cell carcinomas (RCCs) was 40%, which is higher than the range reported in the literature (20–30%). This can be explained by the practice routinely adopted at our center of conducting fewer biopsies and often resorting to surgical intervention. As a result, benign cases that would have been otherwise excluded if a diagnostic biopsy was performed, were included, contributing to the higher proportion of benign tumors in our dataset. A similar proportion between benign SRMs and RCCs was observed by Li et al. (40%) [22], while a lower proportion was observed by Feng et al. (30%) [21], Edirm et al. (25%) [18], Uhlig et al. (20%) [19] and Yu et al. (8%) [17]. Therefore, the differentiation between benign and malignant renal masses suffers from an imbalanced class problem that should be addressed because most ML algorithms require balanced representations of endpoint classes to effectively perform [34,35]. If the imbalance problem is not adequately managed, the classification may be biased towards the majority class and accuracy becomes a misleading metric, thus providing inaccurate results [36].

Our results are in line with several recent studies that have explored the use of radiomics in the characterization of renal masses. Uhlig et al. [19] developed models to distinguish between benign and malignant lesions and tested five different ML algorithms. They found the best performances using Random Forest, which yielded a cross-validated ROC-AUC of 0.83. Kunapuli et al. [15] explored forty features extracted from four-phase contrast-enhanced computed tomography (CECT) images of one hundred and fifty patients with various benign and malignant lesions and reported AUC values of 0.83. Li et al. [22] compared radiomic and clinical models on the validation set, achieving an ROC-AUC equal to 0.84 and 0.76, respectively; in that of Coy et al. [20], oncocytomas and ccRCC were compared and the performance of volume segmentation in the excretory phase showed an accuracy of 74.4%, a sensitivity of 85.8% and a PPV of 80.1%. 

Higher performances have been obtained by some recent studies. Erdim et al. [18] compared eight ML algorithms to construct a prediction model for renal mass diagnosis based on CECT imaging from both benign and malignant lesions, specificity rates and AUC values were reported to be 0.917 and 0.916, respectively, in a patient cohort numerically similar to our study (63 patients). Feng et al. [21] proposed a support vector machine (SVM) model that achieved good accuracy in discriminating between fat-poor AML and RCC in a cohort of 58 patients (AUC of 93.9%), while Yu et al. [17] implemented a SVM algorithm to differentiate oncocytoma from other tumors, obtaining a ROC-AUC of 0.86. Kocak et al. [14] developed a radiomic model for the differentiation between renal cell carcinoma subtypes; their radiomic model achieved high performance both on internal (ROC-AUC = 91.6) and external validation using public datasets (ROC-AUC = 84.6). 

Compared to earlier studies, our study has several strengths. One important consideration is the population of renal masses that have been studied: our radiomic signature was specifically developed for the characterization of small renal masses (<4 cm), as they present the major diagnostic dilemma among renal masses. Indeed, it is well known that size is statistically related to the malignancy of a mass [22], in particular, it has been stated that each 1 cm increase in tumor size is associated with a 16% increase in the odds of malignancy [37] and, as tumors grow in size, other radiologic elements tend to be present, such as necrosis, calcification, pseudocapsule or a central scar, making it easier to suggest a diagnostic hypothesis, even if still huge oncocytomas could pose a diagnostic dilemma. Only considering masses < 4 cm, we excluded from the radiomic signature renal masses that inherently exhibited a high intrinsic malignant potential. The aforementioned size limit was not applied in numerous previous studies that included tumors without any size limit [18,19,38] or were limited to T1 (<7 cm) [19].

Another factor that should be considered in the evaluation of radiomic signatures is the potential variability introduced by different CT scanners. It is essential to assess the robustness of the radiomic signature to ensure its applicability in different clinical settings. Previous studies have typically included one or two different CT scanners, with limited generalizability to other devices (two scanners in Erdim et al. [18], Kocak et al. [14] and Li et al. [22], and one scanner in Sun et al. [38], Kunapuli et al. [15] and Yu et al. [17]). Furthermore, those studies that made use of more than one CT device did not perform a reproducibility analysis or a harmonization strategy. In contrast, our study incorporated data from three different image acquisition devices and included the evaluation of RF robustness in the feature selection strategy. This analysis guarantees the reproducibility of our radiomic signature across a wider range of imaging platforms, enhancing its clinical relevance and potential for broader applications.

A further improvement of the present study concerned the CT clinical protocol used and its applicability in the routine clinical scenario. While the cited studies have reported promising results in the development of radiomic models for renal masses, their algorithms have primarily been developed based on CT scans acquired in multiple phases of contrast medium distribution [39]. However, this approach may limit the applicability and reproducibility of these models in a clinical routine scenario, as the non-enhanced and the arterial phase are not routinely performed, while the venous phase is the most performed in abdomen evaluation, in a real-life approach. Considering that the majority of SRMs are discovered during exams performed for other medical reasons, we decided to develop an algorithm that specifically analyzes lesions segmented on only the venous phase, eliminating the need for further imaging studies to characterize the lesion. In contrast, previous studies, such as those conducted by Erdim et al. [18], Kunapuli et al. [15], Kocak et al. [14] and Feng et al. [21], utilized a standardized multi-phase CT imaging protocol, providing more comprehensive information on mass features that perhaps justify higher diagnostic performances, but potentially reducing the applicability of the radiomic signature to a wider range of clinical scenarios. 

Finally, a strength of our study was the use of a radiomics signature only based on 10 radiomic features. The use of a smaller number of radiomic features allows for a more focused and streamlined analysis, reducing the potential for overfitting and improving the interpretability of our results. By reducing the number of radiomic features, we were able to reduce the complexity of our model and make it more accessible to radiologists and other healthcare professionals, who could use it to improve patient management. Other studies that dealt with the same application included many more features in their models (40 RFs [15], 11–22 RFs [38], 43 RFs [17]).

The current study has certain limitations that should be acknowledged. Firstly, it was retrospectively designed, which can result in inherent disadvantages and data loss. However, it is important to note that most studies that involve CT texture analysis and machine-learning-based algorithms are retrospective in nature. Secondly, the sample size was relatively small due to the strict inclusion criteria that were applied. Nonetheless, these criteria were necessary to ensure correct analysis and to avoid extracting broad features that could have limited specificity.

Another limitation of this study is the lack of a clinical model to be integrated with the radiomic signature, or the implementation of radiometabolomics, transcriptomics or deep learning data. Although the radiomic analysis supplies valuable insights, the implementation of a clinical model and other datasets could enhance the diagnostic utility of the model.

According to the revised WHO classification of renal tumors [40], a subset of entities previously classified as chromophobe renal cell carcinomas have been reclassified as low-grade oncocytic tumors (LOT), falling within the benign spectrum. Regrettably, our study did not account for these updated classifications, representing a limitation in our analysis.

Finally, a last important limitation regards the lack of an external validation that could provide evidence of the model’s generalizability. Further study will be addressed to offset up an independent external dataset for independent validation of the classifiers developed in the study.

## 5. Conclusions

In conclusion, the results of this study prove the feasibility of a radiomic model for the characterization of small renal masses. The use of radiomics in the evaluation of small renal masses has the potential to improve patient management and to facilitate the accurate diagnosis of malignancy.

## Figures and Tables

**Table 1:** Characteristics of small renal masses and patients.

	Histotype	Gender	Age (Years)	Size (mm)
Benign (n = 34)	Lipid poor angiomyolipoma (7)	M = 47.1% F = 52.9%	64 (23)	22.6 (16.4)
	Oncocytoma (25)			
	Renal leiomyoma (2)			
Malignant (n = 51)	Clear cell RCC (37)	M = 68.6% F = 31.4%	67 (13)	28.5 (13.6)
	Chromophobe RCC (7)			
	Papillary RCC (7)			

M: Male; F: Female. Age and size are reported as median and interquartile range.

**Table 2:** Mean performances of the final model with the associated standard deviation of the mean.

	Training Set	Test Set
ROC-AUC	0.79 ± 0.12	0.79 ± 0.04
Accuracy	0.75 ± 0.12	0.73 ± 0.04
Sensitivity	0.77 ± 0.19	0. 78 ± 0.07
Specificity	0.73 ± 0.15	0.63 ± 0.05
PPV	0.82 ± 0.12	0.77 ± 0.06
NPV	0.70 ± 0.17	0.66 ± 0.07
F1 score ^†^	0.71 ± 0.15	0.64 ± 0.08

^†^ The reported F1 score refers to minority class, i.e., benign cases.

## Data Availability

Data are available on request to the corresponding author.

## Supplementary Materials

The following supporting information can be downloaded at: https://www.mdpi.com/article/10.3390/cancers15184565/s1, Figure S1: Flow chart of inclusion and exclusion criteria; Table S1: Histotypes analysis in test set; Table S2: Checklist for Artificial Intelligence in Medical Imaging (CLAIM).
