# Supplementary material for: Small Renal Masses: Developing a Robust Radiomic Signature
Source: Cancers (Basel). 2023 Sep 14;15(18):4565. doi: 10.3390/cancers15184565 (PMC10527518; doi:10.3390/cancers15184565)
Supplement: Supplementary file 1 [file cancers-15-04565-s001.zip › cancers-2548766-supplementary.pdf]

Figure S1. Flow chart of inclusion and exclusion criteria

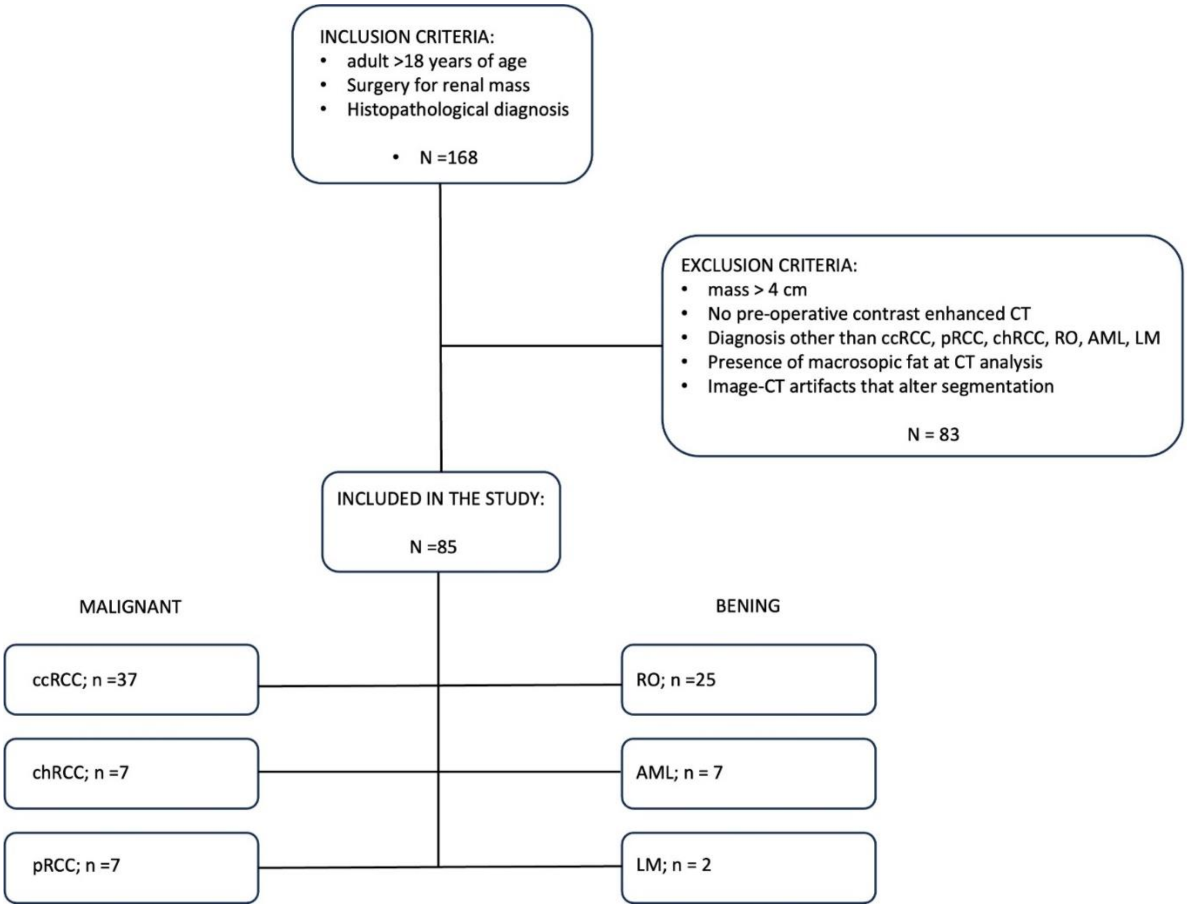

Table S1. Histotypes analysis in test set.

	Histotype	Actual class	predicted class	probability score
16	cc-RCC	malign	malign	0.86
20	p-RCC	malign	malign	0.57
36	p-RCC	malign	malign	0.71
38	ch-RCC	malign	malign	1.00
40	RO	benign	benign	0.29
41	RO	benign	benign	0.43
42	cc-RCC	malign	benign	0.43
44	cc-RCC	malign	benign	0.29
55	cc-RCC	malign	malign	1.00
56	RO	benign	benign	0.43
57	AML	benign	benign	0.29
59	p-RCC	malign	malign	0.57
62	cc-RCC	malign	malign	0.86
63	RO	benign	benign	0.00
65	AML	benign	malign	0.86
74	RO	benign	malign	0.57
82	cc-RCC	malign	malign	0.86
85	cc-RCC	malign	malign	0.71

	correctly classified (%)	wrongly classified (%)
cc-RCC	0.71	0.29
p-RCC	1.00	0.00
ch-RCC	1.00	0.00
RO	0.80	0.20
AML	0.50	0.50
LM	0	0

Table S2. Checklist for Artificial Intelligence in Medical Imaging (CLAIM)

Section/Topic	No.	Item - QUESTIONS
TITLE or ABSTRACT		
	1	Identification as a study of AI methodology, specifying the category of technology used (eg, deep learning)
ABSTRACT		
	2	Structured summary of study design, methods, results, and conclusions
INTRODUCTION		
	3	Scientific and clinical background, including the intended use and clinical role of the AI approach
	4	Study objectives and hypotheses
METHODS		
Study Design	5	Prospective or retrospective study
	6	Study goal, such as model creation, exploratory study, feasibility study, noninferiority trial
Data	7	Data sources
	8	Eligibility criteria: how, where, and when potentially eligible participants or studies were identified (eg, symptoms, results from previous tests, inclusion in registry, patient-care setting, location, dates)
	9	Data preprocessing steps
	10	Selection of data subsets, if applicable
	11	Definitions of data elements, with references to common data elements
	12	De-identification methods
	13	How missing data were handled
Ground Truth	14	Definition of ground truth reference standard, in sufficient detail to allow replication
	15	Rationale for choosing the reference standard (if alternatives exist)
	16	Source of ground truth annotations; qualifications and preparation of annotators
	17	Annotation tools
	18	Measurement of inter- and intrarater variability; methods to mitigate variability and/or resolve discrepancies
Data Partitions	19	Intended sample size and how it was determined
	20	How data were assigned to partitions; specify proportions
	21	Level at which partitions are disjoint (eg, image, study, patient, institution)
Model	22	Detailed description of model, including inputs, outputs, all intermediate layers and connections
	23	Software libraries, frameworks, and packages
	24	Initialization of model parameters (eg, randomization, transfer learning)
Training	25	Details of training approach, including data augmentation, hyperparameters, number of models trained
	26	Method of selecting the final model
	27	Ensembling techniques, if applicable
Evaluation	28	Metrics of model performance
	29	Statistical measures of significance and uncertainty (eg, confidence intervals)
	30	Robustness or sensitivity analysis
	31	Methods for explainability or interpretability (eg, saliency maps) and how they were validated
	32	Validation or testing on external data
RESULTS		
Data	33	Flow of participants or cases, using a diagram to indicate inclusion and exclusion
	34	Demographic and clinical characteristics of cases in each partition
Model performance	35	Performance metrics for optimal model(s) on all data partitions
	36	Estimates of diagnostic accuracy and their precision (such as 95% confidence intervals)
	37	Failure analysis of incorrectly classified cases
DISCUSSION		
	38	Study limitations, including potential bias, statistical uncertainty, and generalizability
	39	Implications for practice, including the intended use and/or clinical role
OTHER INFORMATION		
	40	Registration number and name of registry
	41	Where the full study protocol can be accessed
	42	Sources of funding and other support; role of funders

Checklist for Artificial Intelligence in Medical Imaging (CLAIM)

Section/Topic	No.	Item - **ANSWERS**
TITLE or ABSTRACT		
	1	In the Abstract is reported that the analysis was carried out using machine learning methodology.
ABSTRACT		
	2	The abstracts contains a structured summary of study design, methods, results, and conclusions.
INTRODUCTION		
	3	The introduction contains the relevant scientific and clinical background. It describes how the machine learning model can support the radiological differentiation between benign and malignant small renal masses, improving a diagnostic scenario which is characterized by poor specificity.
	4	The study objective has been anticipated at the end of the introduction section
METHODS		
Study Design	5	The retrospective nature of the study has been specified during the methods section.
	6	The study goal, as the creation of a radiomic model to differentiate benign and malignant small renal masses, is specified in the introduction.
Data	7	The unique data source, i.e. the PACS data archiving system of Parma University Hospital, is specified.
	8	Eligibility criteria are listed in patients subsection of Materials and Methods.
	9	All data preprocessing steps have been described in details. They include redundant RFs elimination, RFs standardization, dataset balancing, exclusion of non-reproducible RFs.
	10	A robust data partition strategy was implemented and described. The subset used in each operation of analysis step has been rigorously reported.
	11	Predictor (radiomic features) and outcome variable (benign vs. malign) have been defined. They represent common variable which had been introduced by the radiology community.
	12	De-identification methods consist in anonymization of CT images and of clinical and radiological data.
	13	Fortunately, no missing data were present.
Ground Truth	14	Ground truth is represented by the radiological differentiation between benign and malignant small renal masses without the use of artificial intelligence, but no measurements of it have been performed.
	15	The theorical performance of the radiological differentiation between benign and malignant small renal masses without the use of artificial intelligence have been derived from literature. Their measurement in our local context can't be performed due to the retrospective nature of the study.
	16	No ground truth data are available.
	17	No annotation tools have been necessary
	18	Inter scanner variability was evaluated. Less repeatable radiomic feature were excluded from the analysis.
Data Partitions	19	The sample size was determined by the enrolling period after the application of exclusion criteria.
	20	Data was divided in training and test considering the Pareto principle (80%-20%), always balancing with respect of the endpoint. Partition details were included in the manuscript.
	21	Level at which partitions are disjoint (eg, image, study, patient, institution)
Model	22	Model construction, its inputs and its outputs are described extensively in the manuscript.
	23	Software libraries, i.e. the Caret and the Unbalance packages of R environment were reported in the manuscript.
	24	Model parameters were optimize manually in a limited range of possible values to maintain model simplicity and to achieve the desired level of explanaibility. Manual selection of model paramiters allows also to avoid the curse of dimensionality problem. Therefore, no initialization values were required.
Training	25	Training details have been reported. They include oversampling of the minority class, feature selection, dimensionality reduction and finally the classifier.
	26	Final model was obtained by maximizing F1 score measure of minority class obtained in the inner validation of training set. The optimization strategy is deeply described in materials and methods section.
	27	No ensambling techniques are used because they are not necessary.
Evaluation	28	Metrics of model performance include ROC-AUC, Accuracy, Specificity, Sensitivity, F1 score, PPV and NPV. They are reported in the manuscript as means and associated standard deviations.
	29	Standard deviations of model performances are reported both for inner validation and test on unsee data. They quantify the stability of the results across runs of Monte Carlo cross validation. In each runs patients included in the training subset were different.
	30	Robustness was achieved by 1-reproducibility analysis of predictors. 2-data partitioning and Monte Carlo Cross-Validation strategy which was iteratively repeated 100 times. The sensitivity analysis, i.e. the stability of model outputs against different training subsets, consisted in the evaluation of performances variability (standard dediviations).
	31	Explainability or interpretability was achieved by using a very simple and understandable model, i.e. KNN. A graphical interpretation of the model is provided.
	32	No external data were available. An independent test on unseen data has been performed.

RESULTS		
Data	33	Flow chart of inclusion and exclusion criteria was included in the supplementary data.
	34	Demographic and clinical characteristics of cases have been reported in table 1. Reporting those characteristics stratifying with respect to partitions was not feasible due to the fact that multiple iterations were performed.
Model performance	35	Performance metrics for optimal model on both training and test sets are reported in table 2-
	36	Estimates of diagnostic accuracy and its standard deviation is provided in table 2.
	37	A rough/tentative failure analysis of incorrectly classified cases is provided in table S1. The possible influence of the histotype (both for malign and bening case) on success or failure of SRM classification was investigated.
DISCUSSION		
	38	Study limitations are listed in the discussion section.
	39	Implications for practice, including the intended use and/or clinical role No practical use of the model is conceivable at the moment. Discussion section contextualizes the strengths of the developed model, including its broader applicability respect to other previous model presented in the litterature.
OTHER INFORMATION		
	40	Registration number and name of registry are reported in the Institutional Review Board Statement section.
	41	The full study protocol data are available on request submitted to the corresponding author.
	42	No funding and other support were available, as stated in the disclosure at the end of the manuscript.
